# Revisiting early angiosperm pollination: a reassessment of *Angimordella* beetle and co-occurring thrips from mid-Cretaceous amber

**DOI:** 10.1186/s12915-026-02572-0

**Published:** 2026-03-27

**Authors:** Yan‑Da Li, David Peris, Constanza Peña-Kairath, Qian Zhao, Di-Ying Huang, Chen-Yang Cai

**Affiliations:** 1 State Key Laboratory of Palaeobiology and Stratigraphy, Nanjing Institute of Geology and Palaeontology, Chinese Academy of Sciences, Nanjing, 210008 China; 2 Bristol Palaeobiology Group, School of Earth Sciences, University of Bristol, Bristol, BS8 1TQ UK; 3 Institut Botànic de Barcelona (CSIC-CMCNB), Barcelona, 08038 Spain; 4 Núcleo de Estudios Transdisciplinarios del Cuaternario del Sur de Chile (TAQUACh), Universidad Austral de Chile, Valdivia, Chile

**Keywords:** Kachin amber, Angiosperm pollination, Mordellidae, Thrips

## Abstract

**Background:**

The Cretaceous rise of angiosperms profoundly reshaped terrestrial ecosystems, yet direct fossil evidence of early insect–angiosperm interactions remains scarce. Here, we re-examine a key mid-Cretaceous amber inclusion from Kachin, Myanmar, previously reported to preserve a pollen-bearing beetle (*Angimordella burmitina*) in close association with eudicot pollen.

**Results:**

Our reassessment supports the recent transfer of *Angimordella* from Mordellinae (crown-group Mordellidae) to Apotomourinae (stem-group Mordellidae), based on traits such as a short pygidium and the absence of a subapical metatibial ridge. This revised placement challenges earlier interpretations of *Angimordella* as a specialized angiosperm pollinator and instead suggests a more generalized or transitional ecological role. We also document a co-preserved thrips in direct contact with the pollen grains, representing the first fossil record of a thrips associated with angiosperm pollen.

**Conclusions:**

This fossil assemblage sheds light on the ecological complexity of mid-Cretaceous pollination systems, offering new insights into the incremental development of structured angiosperm–insect pollination networks.

**Supplementary Information:**

The online version contains supplementary material available at 10.1186/s12915-026-02572-0.

## Background

The macroecological revolution triggered by the Cretaceous rise of angiosperms represents one of the most profound ecological and evolutionary transitions in Earth’s history [1]. Insect-mediated pollination is considered one of the key drivers of this diversification. This mutualistic interaction may have stimulated rapid evolutionary changes in both angiosperms and their insect partners [2]. Direct fossil evidence of insect–angiosperm interactions during this critical period remains exceptionally rare, yet it is vital for understanding the coevolutionary dynamics that shaped modern pollination networks [3–6].

While often overshadowed by bees and butterflies, beetles represent crucial flower visitors and pollinators, particularly in certain environments such as tropical forests [7–9]. Fossil beetles from Cretaceous amber deposits have provided key insights into early pollination systems. For example, members of Oedemeridae in Lower Cretaceous Spanish amber and Boganiidae in mid-Cretaceous Kachin amber have been associated with gymnosperm pollination, with the boganiid *Cretoparacucujus* Cai and Escalona also exhibiting specialized morphological adaptations for pollen feeding [10, 11]. Additionally, several species of Kateretidae/Nitidulidae have been discovered in Kachin amber, with some individuals associated with gymnosperms and others with angiosperms [4, 12–14].

Thrips, despite their small size, limited flight capacity, and lack of specialized pollen-carrying structures, constitute another underappreciated group of pollinators [15–17]. Recent studies have begun to uncover their importance in the early evolution of insect pollination [18–20]. Notably, flower-visiting thrips may have even facilitated the transition of bees to pollinivory by serving as prey for early bee ancestors [21]. Fossil thrips from Cretaceous amber, such as Melanthripidae and Thripidae in Spanish amber, Stenurothripidae in Kachin and Tilin ambers, and stem-group Phlaeothripidae in Kachin amber, have been associated with gymnosperm pollination [18–20, 22, 23]. However, their extant relatives are mostly linked to angiosperms, suggesting a likely host shift from gymnosperms to angiosperms [19].

**Fig. 1:**
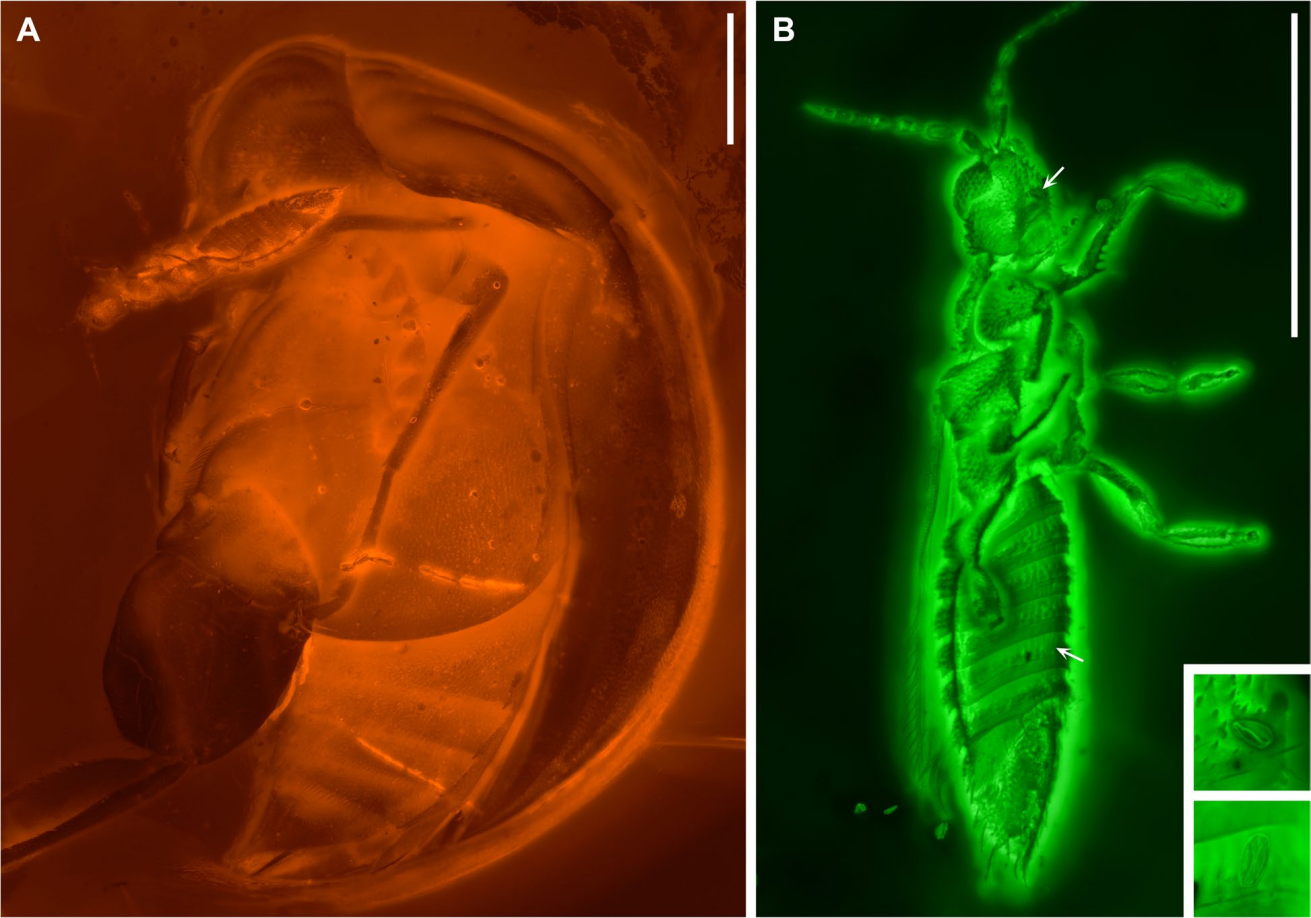
Association of *Angimordella* beetle, thrips, and eudicot pollen in mid-Cretaceous amber (NIGP171315). **A** *Angimordella burmitina* preserved together with a thrips, under widefield fluorescence. **B** Thrips under confocal microscopy, with arrows indicating two pollen grains in direct contact with the thrips (close-ups in insets). Scale bars: 500 µm

Bao et al. [24] reported a mordellid beetle (*Angimordella burmitina* Bao et al.) with eudicot pollen in contact with different body parts in mid-Cretaceous Kachin amber (Fig. 1A), which they interpreted as direct evidence of specialized angiosperm pollination. However, the taxonomic placement of the beetle within Mordellinae (crown-group Mordellidae) is incorrect [25], a misclassification that directly affects inferences about its pollination ecology. In addition, the same amber piece preserved a thrips that was overlooked (Fig. 1B), leaving its potential ecological significance unexplored.

Although Batelka et al. [25] have transferred *Angimordella* Bao et al. to Apotomourinae, this reassignment was made without detailed justification or discussion of diagnostic characters. In the present study, we re-examine the specimen described by Bao et al. [24], corroborating the placement of *Angimordella* in Apotomourinae, a subfamily of stem-group Mordellidae. This revised classification prompts a re-evaluation of the beetle’s ecological role in early pollination systems. We also document the co-preserved thrips, which, despite limited morphological preservation, represent the first documented case of a Cretaceous thrips in direct contact with angiosperm pollen. Together, these findings provide new insights into the structure and evolution of early angiosperm pollination networks during the Cretaceous.

## Results and discussion

### *Angimordella* belongs to Apotomourinae, not crown Mordellidae

**Fig. 2:**
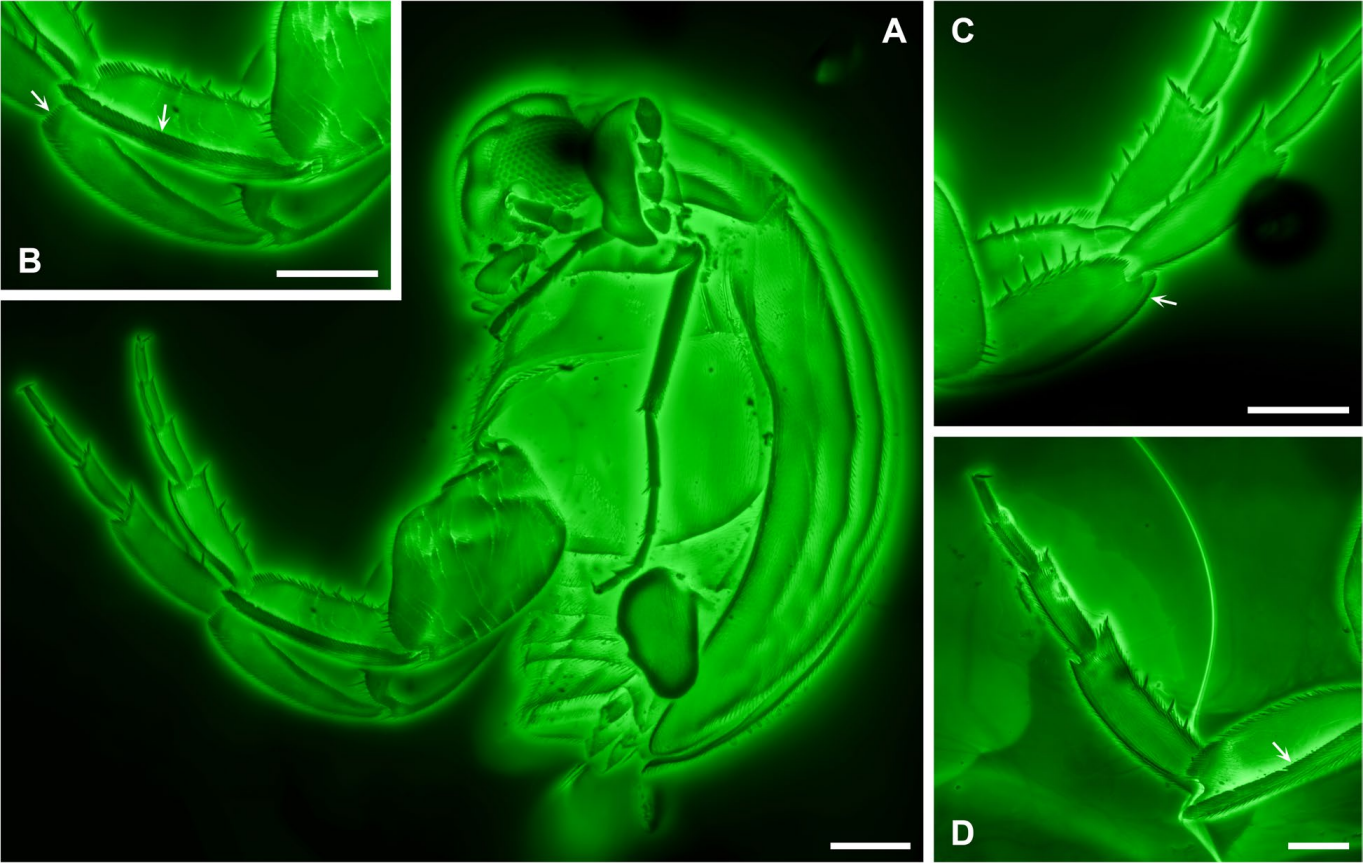
Morphological details of *Apotomoura fortiscrura* and *Angimordella burmitina* under confocal microscopy. **A** *A. fortiscrura* (NIGP168214), lateral habitus. **B**, **C** Hind legs of *A. fortiscrura* (NIGP168214), with arrows indicating the apical metatibial spine ridge, which curves and further extends longitudinally. **D** Hind leg of *A. burmitina* (NIGP171315), with arrow indicating the apical metatibial spine ridge, similarly curving and extending longitudinally. Scale bars: 200 µm

**Fig. 3:**
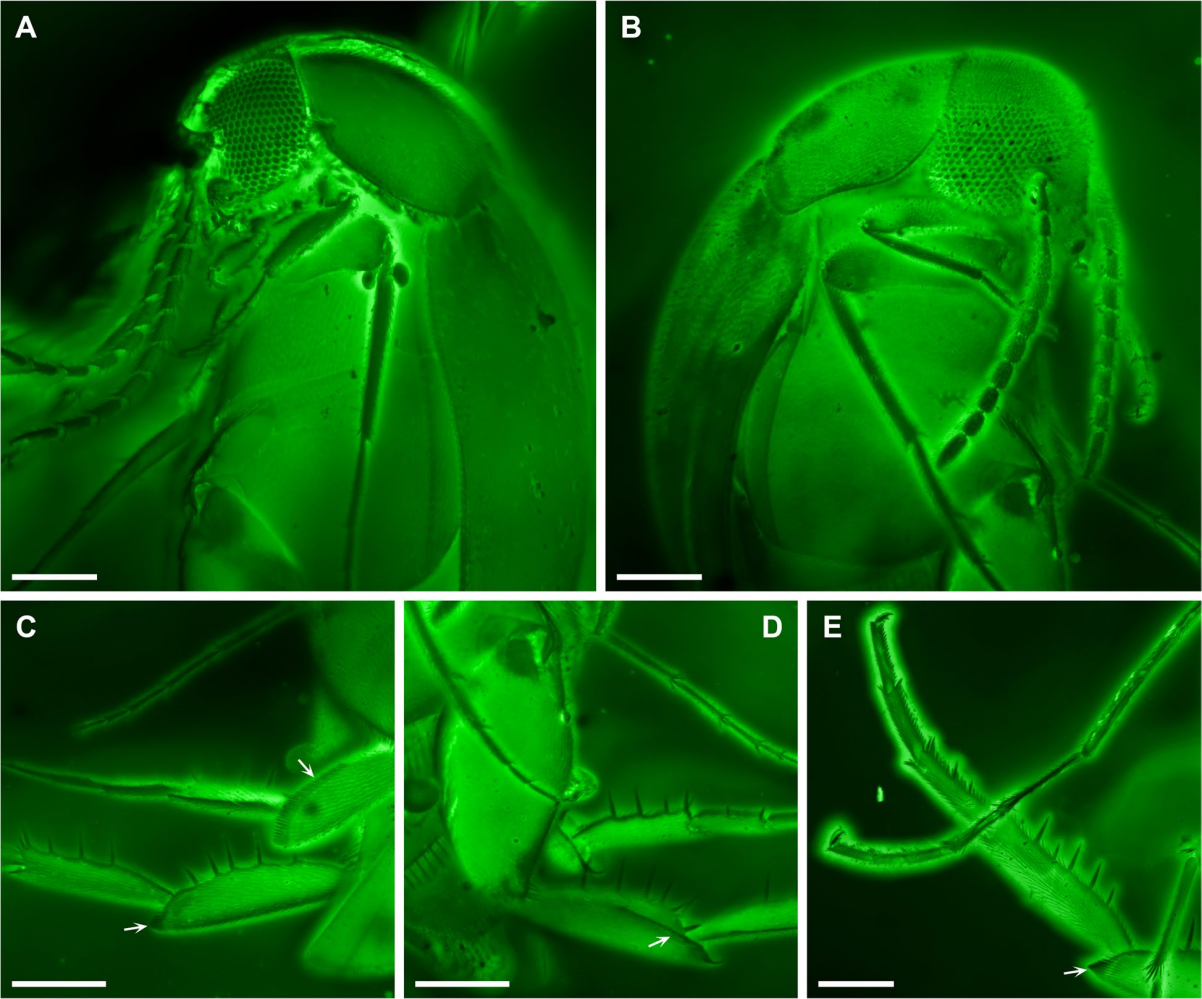
Morphological details of *Multispinus multispinosus* under confocal microscopy. **A** Anterior body of NIGP168210. **B** Anterior body of NIGP168212. **C**–**E** Hind legs of NIGP168212 (**C**, **D**) and NIGP168211 (**E**), with arrows indicating the apical metatibial spine ridge, which curves and further extends longitudinally. Scale bars: 200 µm

Bao et al. [24] placed the extinct genus *Angimordella* within the extant subfamily Mordellinae of crown-group Mordellidae. However, their justification for this classification is questionable. In particular, they listed a “very short pygidium” (Fig. 1A) as a supporting character for assigning *Angimordella* to Mordellinae. This is problematic because all extant members of Mordellidae possess an elongate pygidium. In contrast, a very short pygidium is characteristic of Apotomourinae (Fig. 2A) [26, 27]. Furthermore, Bao et al. [24] claimed that *Angimordella* resembles *Primaevomordellida* Bao et al. from Burmese amber in lacking a ridge on the metatibia. This assertion is incorrect and misleading. In fact, *Primaevomordellida*, as a true member of Mordellinae, exhibits distinct apical and subapical spine ridges on the metatibia [25]. In contrast, the metatibial spine ridge of *Angimordella* is highly unusual and differs from that of extant Mordellidae but is instead consistent with the condition in Apotomourinae (Figs. 2B–D, 3C–E). In extant Mordellidae (except for *Reynoldsiella* Ray), the metatibia bears not only an apical spine ridge at its distal end but also at least one subapical ridge [28]. This subapical ridge may run parallel to the apical margin of the tibia or curve and further extend along the longitudinal axis of the tibia. However, in *Angimordella* and other members of Apotomourinae, the subapical ridge is absent, while the apical ridge curves and further extends longitudinally (Figs. 2B–D, 3C–E). Additionally, both *Angimordella* and Apotomourinae exhibit isolated upright spines on the metatibia and metatarsi, which are not seen in extant Mordellidae.

The short pygidium and absence of a subapical metatibial ridge in Apotomourinae are probably plesiomorphic traits, as both conditions are widespread in close relatives of Mordellidae, especially Ripiphoridae, which suggests that Apotomourinae may represent a stem group of Mordellidae. Apotomourinae previously included two genera, *Multispinus* Bao et al. and *Apotomoura* Bao et al., which differ in the arrangement of metatibial spines and antennal morphology. In the phylogenetic analysis of Batelka et al. [25], *Angimordella* appeared basal within Apotomourinae rather than clustering closely with *Apotomoura*, likely due to an incorrect scoring of ventral metatibial spines as absent. In fact, *Angimordella* is remarkably similar to *Apotomoura*, differing primarily in its larger body size, and we have not identified any definitive distinguishing features between them thus far. However, we refrain from making any taxonomic revisions at this time, leaving such actions to future, more comprehensive studies.

### A snapshot of emerging angiosperm pollination networks

The exceptional co-preservation of a stem-mordellid beetle (*Angimordella*), a thrips, and eudicot pollen grains in contact with both insects (Fig. 1B; fig. 2D, E in [24]), holds significant ecological implications. Although the possibility that the pollen was produced by Erdtmanithecales gymnosperms cannot be entirely excluded, the tricolpate appearance of the preserved pollen, as noted by Bao et al. [24] and Tihelka et al. [29], is most consistent with eudicot angiosperms. Therefore, this specimen provides important insights into the ecological dynamics of insect-mediated pollination during the diversification of angiosperms.

Modern mordellids exhibit specialized pollen-feeding behaviors linked to angiosperm pollination [30], whereas their close relatives, the ripiphorids, display different ecological strategies [31]. While ripiphorid larvae are parasitoids, adults of some groups are associated with blossoms of flowering plants, where they lay their eggs, facilitating larval parasitism of other flower-visiting insects [32]. Additionally, some adult ripiphorids may feed on nectar. Although no specialized pollination behavior has been reported for ripiphorids, their brood-site association with flowers likely increases the chance of incidental pollen transfer during extended floral visits.

Bao et al. [24] placed *Angimordella* within Mordellinae (crown-group Mordellidae) and proposed that it functioned as a specialized flower-visiting pollinator, similar to extant mordellids. However, the reclassification of *Angimordella* within the subfamily Apotomourinae (stem-group Mordellidae), rather than crown-group Mordellidae, challenges the direct comparisons with extant pollen-feeding specialists. This revised phylogenetic placement situates *Angimordella* at a pivotal position within the Ripiphoridae–Mordellidae clade, possibly representing an intermediate stage in the evolutionary transition from the brood-site floral association seen in some Ripiphoridae (where flower visitation is primarily driven by oviposition) towards the more obligate pollinivory and pollination specialization characteristic of crown Mordellidae (see also [33] for an analogous transition). Although Bao et al. [24] argued that *Angimordella* possesses an enlarged apical maxillary palpomere that may have facilitated pollen collection and transport, this structure is only moderately expanded and not strongly securiform. Comparable moderate expansions of the maxillary palpomere are found in certain ripiphorids that are probably unrelated to flower visiting (e.g., [34]; fig. 106c in [35]). The absence of unequivocal pollen-feeding adaptations in *Angimordella* also suggests that its interaction with contemporaneous angiosperms may have been occasional rather than obligate.

The co-preserved thrips also carries important implications. Although its precise taxonomic placement remains uncertain due to the limited viewing angle (Additional file 1: Text S1, Fig. S1), this specimen represents the first fossil record of a thrips in direct association with angiosperm pollen [19]. In the absence of specialized morphological features associated with pollinivory, the nature of this association remains ambiguous [36]. Nevertheless, extant pollinating thrips generally lack specialized structures for carrying pollen [15], and pollen grains may attach rather randomly to various parts of their bodies [16, 37]. Thus, even without definitive evidence of pollen feeding or active pollination, the co-occurrence of a thrips and eudicot-type pollen in this context may indicate some form of pollination relationship. This fossil therefore adds a new data point to the sparse fossil record of early insect–angiosperm interactions, extending the potential ecological roles of Mesozoic thrips beyond their previously documented associations with gymnosperms (e.g., [18, 19]). It underscores the importance of documenting such interactions to better understand the range of ecological scenarios that may have existed during the emergence of angiosperm pollination systems.

Collectively, the beetle–thrips–angiosperm pollen assemblage offers a rare glimpse into the ecological complexity of early insect–angiosperm interactions during a critical phase of angiosperm evolution. Pollination research on extant angiosperms has recognized that flowers fall along a spectrum from specialist (relying on a narrow set of specific pollinators) to generalist (attracting diverse visitors) syndromes [38]. In particular, flowers visited by beetles or thrips often align with generalist syndromes, in which multiple orders of insects can act as pollen carriers, albeit with varying efficiencies [8, 38]. Thus, given that neither insect in the present fossil assemblage exhibits definitive adaptations for specialized pollen consumption or pollination, their co-occurrence with eudicot-type pollen is consistent with a mixed-visitor or generalist pollination context. This fossil snapshot therefore captures an early chapter in the coevolutionary history of flowering plants and their insect visitors, illustrating how diverse, potentially non-specialized visitors may have participated in the emergence of angiosperm pollination networks.

## Conclusions

The present reassessment not only refines the taxonomic placement of the fossil mordellid but also illuminates the ecological complexity of Cretaceous pollination systems. While earlier studies have primarily focused on transitional stages involving a gymnosperm-to-angiosperm host shift among pollinators [4, 10, 19], the coexistence of the beetle, thrips, and eudicot-type pollen in the present specimen highlights another dimension of transitional insect–angiosperm interactions, offering insights into ecological scenarios that may have preceded the establishment of mutualistic specialization in pollination systems. By integrating these previously insufficiently explored elements, our study advances understanding of the incremental processes that underpinned the emergence of structured pollination networks during angiosperm diversification.

## Methods

The published Kachin amber specimens of *Angimordella burmitina* (NIGP171315), *Apotomoura fortiscrura* Bao et al. (NIGP168213, NIGP168214) and *Multispinus multispinosus* Bao et al. (NIGP168210, NIGP168211, NIGP168212) were re-examined (Figs. 1, 2, and 3). These specimens originated from amber mines near Noije Bum (26° 20′ N, 96° 36′ E), Hukawng Valley, Kachin State, northern Myanmar, and are deposited in the Nanjing Institute of Geology and Palaeontology, Chinese Academy of Sciences, Nanjing, China.

Widefield fluorescence images were obtained with a Zeiss Axio Imager 2 light microscope combined with a fluorescence imaging system. Confocal images were obtained with a Zeiss LSM710 confocal laser scanning microscope, using the 488 nm (Argon) laser excitation line [39]. Widefield fluorescence images were stacked with Helicon Focus 7.0.2 and Zerene Stacker 1.04. Confocal images were stacked with Helicon Focus 7.0.2 and Adobe Photoshop CC. Images were further processed in Adobe Photoshop CC to adjust brightness and contrast.

## Supplementary Information

Additional file 1. Text S1. Remarks to the thrips. Fig. S1. Thrips in Kachin amber specimen NIGP171315, under confocal microscopy.

## Data Availability

All fossils discussed in the paper are reposited in a publicly accessible institution. The original confocal data are available in the Zenodo repository (10.5281/zenodo.17516364).

## References

[1] Benton MJ, Wilf P, Sauquet H. The angiosperm terrestrial revolution and the origins of modern biodiversity. New Phytol. 2022;233(5):2017–35. 34699613 10.1111/nph.17822

[2] Peris D, Condamine FL. The angiosperm radiation played a dual role in the diversification of insects and insect pollinators. Nat Commun. 2024;15(1):552. 38253644 10.1038/s41467-024-44784-4 PMC10803743

[3] Grimaldi DA, Peñalver E, Barrón E, Herhold HW, Engel MS. Direct evidence for eudicot pollen-feeding in a Cretaceous stinging wasp (Angiospermae; Hymenoptera, Aculeata) preserved in Burmese amber. Commun Biol. 2019;2(1):408. 31728419 10.1038/s42003-019-0652-7 PMC6838090

[4] Peris D, Labandeira CC, Barron E, Delclos X, Rust J, Wang B. Generalist pollen-feeding beetles during the mid-Cretaceous. iScience. 2020;23(3):100913. 32191877 10.1016/j.isci.2020.100913 PMC7113562

[5] Peris D, Ollerton J, Sauquet H, Hidalgo O, Peñalver E, Magrach A, et al. Evolutionary implications of a deep‐time perspective on insect pollination. Biol Rev. 2025;100:1452–66. 40070008 10.1111/brv.70008 PMC12227787

[6] Peña-Kairath C, Delclòs X, Álvarez-Parra S, Peñalver E, Engel MS, Ollerton J, et al. Insect pollination in deep time. Trends Ecol Evol. 2023;38(8):749–59. 37062597 10.1016/j.tree.2023.03.008

[7] de Meiros BAS, Peris D. The evolution of flower beetles as visitors and pollinators. Annu Rev Entomol. 2026;71:557–75. 41218280 10.1146/annurev-ento-121423-013404

[8] Bernhardt P. Convergent evolution and adaptive radiation of beetle-pollinated angiosperms. Plant Syst Evol. 2000;222:293–320.

[9] Muinde J, Katumo DM. Beyond bees and butterflies: the role of beetles in pollination system. J Nat Conserv. 2024;77:126523.

[10] Peris D, Labandeira CC, Peñalver E, Delclòs X, Barrón E, de la Pérez- Fuente R. The case of *Darwinylus marcosi* (Insecta: Coleoptera: Oedemeridae): a Cretaceous shift from a gymnosperm to an angiosperm pollinator mutualism. Commun Integr Biol. 2017;10(4):897–904.

[11] Cai C, Escalona HE, Li L, Yin Z, Huang D, Engel MS. Beetle pollination of cycads in the Mesozoic. Curr Biol. 2018;28(17):2806–12. 30122529 10.1016/j.cub.2018.06.036

[12] Peris D, Jelínek J, Sabatelli S, Liu M-K, Peña-Kairath C, Zhao Q, et al. Archaic sap beetles (Coleoptera: Nitidulidae) as Cretaceous pollinators. Palaeoentomology. 2024;7(5):594–610.

[13] Tihelka E, Li L, Fu Y, Su Y, Huang D, Cai C. Angiosperm pollinivory in a Cretaceous beetle. Nat Plants. 2021;7(4):445–51. 33846595 10.1038/s41477-021-00893-2

[14] Zhao Q, Sun Y, Liu J, Ślipiński A, Fu Y, Huang D, et al. Antennal extremes in amber: possible beetle pollination of Chloranthaceae in the Cretaceous. Insect Syst Divers. 2026;10(1):ixaf059.

[15] Terry I. Thrips: the primeval pollinators. In: Marullo R, Mound L, editors. Thrips and tospoviruses: Proceedings of the 7th International Symposium on Thysanoptera. Canberra: Australian National Insect Collection; 2002. p. 157–62.

[16] Varatharajan R, Maisnam S, Shimray CV, Rachana RR. Pollination potential of thrips (Insecta: Thysanoptera)–an overview. Zoo’s Print. 2016;31(4):6–12.

[17] Pop C, Terry I, Mound LA, van der Kooi CJ. Tiny but significant: on the importance of thrips as pollinators. Ann Bot. 2025;136(4):669–82. 10.1093/aob/mcaf069 PMC12464953 40243278

[18] Peñalver E, Labandeira CC, Barrón E, Delclòs X, Nel P, Nel A, et al. Thrips pollination of Mesozoic gymnosperms. Proc Natl Acad Sci USA. 2012;109(22):8623–8. 22615414 10.1073/pnas.1120499109 PMC3365147

[19] Peñalver E, Peña-Kairath C, Barrón E, Nel P, Nel A, Delclòs X, et al. Diverse Mesozoic thrips carrying pollen during the gymnosperm-to-angiosperm plant-host ecological shift. iScience. 2025;28(4):112108. 40248120 10.1016/j.isci.2025.112108 PMC12005343

[20] Peña-Kairath C, Peñalver E, Peris D, Delclòs X. Swarming behaviour and pollination by Cretaceous thrips (Insecta: Thysanoptera). Palaeoentomology. 2024;7(6):723–39.

[21] Sann M, Niehuis O, Peters RS, Mayer C, Kozlov A, Podsiadlowski L, et al. Phylogenomic analysis of Apoidea sheds new light on the sister group of bees. BMC Evol Biol. 2018;18:71. 29776336 10.1186/s12862-018-1155-8 PMC5960199

[22] Guo D, Engel MS, Shih C, Ren D. New stenurothripid thrips from mid-Cretaceous Kachin amber (Thysanoptera, Stenurothripidae). ZooKeys. 2024;1192:197–212. 38425444 10.3897/zookeys.1192.117754 PMC10902785

[23] Ulitzka MR. Late Cretaceous thrips (Thysanoptera) from Hti Lin amber. Zootaxa. 2024;5489(1):99–106. 39646810 10.11646/zootaxa.5489.1.6

[24] Bao T, Wang B, Li J, Dilcher D. Pollination of Cretaceous flowers. Proc Natl Acad Sci USA. 2019;116(49):24707–11. 31712419 10.1073/pnas.1916186116 PMC6900596

[25] Batelka J, Kundrata R, Straka J. Phylogenomics and revised classification of Lymexyloidea and Tenebrionoidea (Coleoptera: Polyphaga: Cucujiformia). Syst Entomol. 2025;50:794–812.

[26] Bao T, Walczyńska KS, Moody S, Wang B, Rust J. New family Apotomouridae fam. nov. (Coleoptera: Tenebrionoidea) from lower Cenomanian amber of Myanmar. Cretac Res. 2018;91:14–9.

[27] Bao T. A new small-bodied mordellid beetle (Coleoptera: Mordellidae) from mid-Cretaceous Burmese amber and taxonomic revision. Acta Palaeontol Sin. 2020;59(1):112–8.

[28] Franciscolo ME. Coleoptera: Mordellidae. A monograph of the South African genera and species: 1. Morphology, subfamily Ctenidiinae and tribe Stenaliini. In: Hanström B, Brinck P, Rudebeck G, editors. South African animal life: results of the Lund University expedition in 1950–1951, Vol 4. Stockholm: Almqvist & Wiksell; 1957. p. 207–291.

[29] Tihelka E, Li L, Fu Y, Su Y, Huang D, Cai C. Reply to: *Pelretes vivificus* was a pollinator of Cretaceous angiosperms. Nat Plants. 2022;8(1):41–4. 34949806 10.1038/s41477-021-01045-2

[30] Lawrence JF, Ślipinśki A. Mordellidae Latreille, 1802. In: Leschen RA, Beutel RG, Lawrence JF, editors. Handbook of Zoology, Arthropoda: Insecta, Coleoptera, beetles, Vol 2: morphology and systematics (Elateroidea, Bostrichiformia, Cucujiformia partim). Berlin: Walter de Gruyter; 2010. p. 533–7.

[31] Lawrence JF, Falin ZH, Ślipinśki A. Ripiphoridae Gemminger and Harold, 1870 (Gerstaecker, 1855). In: Leschen RA, Beutel RG, Lawrence JF, editors. Handbook of Zoology, Arthropoda: Insecta, Coleoptera, beetles, Vol 2: morphology and systematics (Elateroidea, Bostrichiformia, Cucujiformia partim). Berlin: Walter de Gruyter; 2010. p. 538–48.

[32] Vaurie P. A review of the genus *Macrosiagon* in Mexico, with notes on *Rhipiphorus* (Coleoptera, Rhipiphoridae). Am Mus Novit. 1955;1717:1–19.

[33] Ishida C, Kono M, Sakai S. A new pollination system: brood-site pollination by flower bugs in *Macaranga* (Euphorbiaceae). Ann Bot. 2009;103(1):39–44. 18996950 10.1093/aob/mcn212 PMC2707287

[34] Falin ZH. Ripiphoridae Gemminger and Harold 1870 (1853). In: Arnett RH, Thomas MC, Skelley PE, Frank JH, editors. American Beetles, Vol 2: Polyphaga: Scarabaeoidea through Curculionoidea. Boca Raton, FL: CRC Press; 2002. p. 431–44.

[35] Falin ZH. Phylogenetic analysis and revision of the genera and subfamilies of the Ripiphoridae (Coleoptera). Lawrence, KS: University of Kansas; 2003.

[36] Heming BS. Structure, function, ontogeny, and evolution of feeding in thrips (Thysanoptera). In: Schaefer CW, Leschen RA, editors. Functional morphology of insect feeding. Lanham, MD: Thomas Say Publications in Entomology, Entomological Society of America; 1993. p. 3–41.

[37] Eliyahu D, McCall AC, Lauck M, Trakhtenbrot A, Bronstein JL. Minute pollinators: the role of thrips (Thysanoptera) as pollinators of pointleaf manzanita, *Arctostaphylos pungens* (Ericaceae). J Pollinat Ecol. 2015;16:64–71. 26207155 PMC4509684

[38] Willmer P. Pollination and floral ecology. Princeton, NJ: Princeton University Press; 2011.

[39] Fu YZ, Li YD, Su YT, Cai CY, Huang DY. Application of confocal laser scanning microscopy to the study of amber bioinclusions. Palaeoentomology. 2021;4(3):266–78.

