# Supplementary material for: Revisiting early angiosperm pollination: a reassessment of Angimordella beetle and co-occurring thrips from mid-Cretaceous amber
Source: BMC Biol. 2026 Mar 27;24:111. doi: 10.1186/s12915-026-02572-0 (PMC13147661; doi:10.1186/s12915-026-02572-0)
Supplement: Supplementary file 1 — Additional file 1. Text S1. Remarks to the thrips. Fig. S1. Thrips in Kachin amber specimen NIGP171315, under confocal microscopy. [file 12915_2026_2572_MOESM1_ESM.pdf]

Supplementary Information for

# **Revisiting early angiosperm pollination: a reassessment of *Angimordella* beetle and co-occurring thrips from mid-Cretaceous amber**

Yan-Da Li, David Peris, Constanza Peña-Kairath, Qian Zhao, Di-Ying Huang, Chen-Yang Cai

*BMC Biology*
DOI: 10.1186/s12915-026-02572-0

**Text S1. Remarks to the thrips**

This insect can be readily identified as a thrips (order Thysanoptera) based on its overall appearance, particularly the presence of fringed wings, a posteriorly directed mouthcone, and legs terminating in a bladder-like arolium. It can be further assigned to the suborder Terebrantia due to the shape of the terminal abdominal segment, which is conical rather than elongated and tube-shaped as in Tubulifera.

However, the antennal sensillar pattern and wing venation cannot be clearly observed, and these characters are essential for assigning a thysanopteran specimen to a specific family. Consequently, a confident family-level placement is not feasible. The dorsal surfacec of its head shows a reticulated sculpture that differs from all five formally described species of Terebrantia from Kachin amber, suggesting that this specimen likely represents an undescribed species.

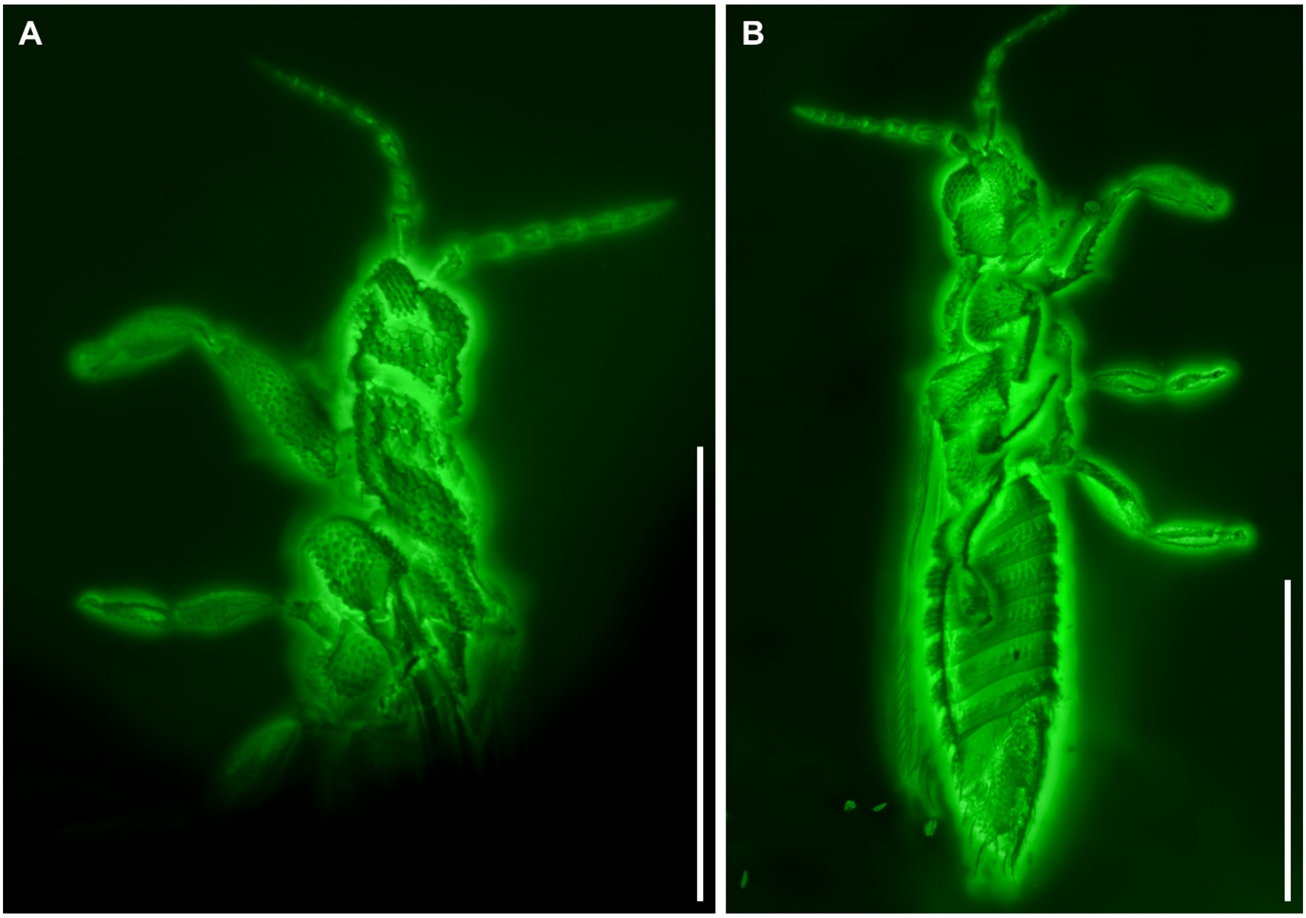


**Fig. S1.** Thrips in Kachin amber specimen NIGP171315, under confocal microscopy. **A**, Dorsal view. **B**, Ventral view. Scale bars: 500 μm.
